# Coming Up Next: The Extent of the Perceptual Window in Comic Reading

**DOI:** 10.1111/cogs.70142

**Published:** 2025-11-19

**Authors:** Clare Kirtley, Christopher Murray, Phillip B. Vaughan, Benjamin W. Tatler

**Affiliations:** ^1^ School of Psychology University of Aberdeen; ^2^ School of Humanities University of Dundee; ^3^ Department of Games Technology and Mathematics Abertay University

**Keywords:** Comics, Visual narratives, Eye‐tracking, Peripheral information

## Abstract

Recent models of sequential narratives suggest that readers form predictions about upcoming panels as they read. However, previous work has considered these predictions only in terms of currently viewed information. In the current studies, we investigate to what extent readers are using information from un‐fixated panels in comic stories. Using the moving‐window paradigm, we studied whether reading behavior was disrupted when upcoming panels were unavailable to the reader, in short comic strips (Experiment 1) and multipage comics (Experiment 2). Both studies showed the greatest disruption to reading when all peripheral information was removed, but such changes persisted when only partial peripheral information was available. The results indicate that readers are making use of information from at least two panels ahead of the current fixation location. We consider these findings in relation to the PINS model of comic reading, and how the role of peripheral information might be further explored.

## 1 Introduction

Word‐image combinations are a common feature of everyday life, used to convey information in adverts, newspapers, textbooks, and many other sources. Given the frequency with which we encounter these items, it becomes important to understand more about them, and how readers take information from multiple sources in order to arrive at an understanding. Meyer's multimedia theory (e.g., Meyer, 2002) explores how readers’ comprehension of a subject is increased when they study it using materials that incorporate both text and image, and previous eye‐tracking work has quantified the typical exploration patterns of a reader encountering these sources (e.g., Carroll, Young, & Guertin, 1992; Rayner, Rotello, Stewart, Keir, & Duffy, 2001).

Much of the initial work into multimodal stimuli focused on self‐contained examples, where the text‐image combination presented had been designed to stand alone (e.g., textbooks, where a single diagram typically illustrates a longer passage of text). However, in recent years, more work has been carried out on how readers understand sequential visual narratives, such as films (e.g., Loschky, Hutson, Smith, Smith, & Magliano, 2018; 2020) and comics (e.g., Cohn, 2013a). In comics, the story is presented in a series of panels, each providing a static image, often, but not always, including text, from which the reader must gather and integrate information and understand how panel contents combine to provide a coherent narrative.

Given the necessarily sequential nature of overt inspection while reading comics—we can only direct the fovea to one panel at a time—and the sequential narrative conveyed in a comic, it is unsurprising that many authors have drawn comparisons between process of reading a comic strip and that of reading a sentence (Cohn, 2013a; Foulsham, Wybrow, & Cohn, 2016; Kirtley, Murray, Vaughan, & Tatler, 2018). Interestingly, early theoretical approaches to comic reading and comprehension rejected the idea of strong similarities to text reading. It was suggested that such constructions had no grammar or set of rules to guide the reader (Postema, 2013). Comprehension was seen as a holistic process, via integration of all the information on the page (Barber, 2002), with the related eye movements appearing erratic (Groensteen, 2007; Miodrag, 2013). However, other authors have challenged both the notion that comics are constructed without “grammar” or rules and that the reader's processing is holistic and inspection erratic. For example, other work has suggested that comprehension may be both holistic and piecemeal. Hatfield (2009) proposes that a key part of the comic experience is the “tension” between how the reader may choose to view a page—as a series of individual panels, each illustrating a moment in a sequence, or as a combination of all the panels on the page, making up the overall layout.

Evidence for readers’ sensitivity to such organization within comics continues to grow. If readers gathered information holistically from all panels simultaneously (or if comics truly had no underlying rule system), then disruptions to the order of panels in the comic should not affect the reader. Yet, when studies have manipulated the narrative sequence depicted across panels, for example, by changing the order (Foulsham et al., 2016) or deleting panels (Cohn, 2014), readers have been found to have difficulty in understanding the new arrangement. Foulsham et al. (2016) recorded the eye movements of participants while reading strips with scrambled or correct panel ordering and noted that readers made more fixations and regressive saccades when engaging with the scrambled versions. Eye‐tracking studies such as those by Foulsham et al. (2016) and Kirtley et al. (2018) show that the progression of the eye through the page is not erratic, but largely follows the sequence of panels in a standard order.

Cohn (e.g., Cohn, 2014; 2014b; Cohn, Jackendoff, Holcomb, & Kuperberg, 2014) suggests that comics are written in a “visual language,” in which comprehension of the sequence involves understanding the composition of the images (graphic structure), understanding how these images fit together narratively (narrative structure), and how the panels containing the images are arranged on the page (external compositional structure). More recently, two complementary models for reading visual narrative sequences have been proposed to formalize the process: the Scene Perception and Event Comprehension Theory (SPECT, Loschky et al., 2018; Loschky, Larson, Smith, & Magliano, 2020) Model and the Parallel Interfacing Narrative‐Semantics (PINS) Model (Cohn, 2020b).

The SPECT model draws on perceptual and attentional evidence, and suggests there are two key processes—front‐end and back‐end processes. The PINS model uses neurocognitive evidence from electroencephalography (EEG) studies and suggests there are two information pathways—semantic and narrative. Both models provide a similar overview of the processing of image sequences: information is gathered from the current part of the visual sequence (e.g., a comic panel) and used to allow the reader to construct a situation model of the narrative. The reader can use the situation model to make predictions as to likely upcoming events in the narrative. As the reader reads on, more information is acquired, confirming or not the predictions made, and causing the model to be updated (Cohn, 2020a).

SPECT's front‐end processes are the information‐gathering stage. The reader attends to the informative regions of the comic panel, and extracts key information from the fixations they make on these locations. Previous work has found that readers are typically focused on highly informative panel regions (e.g., the faces of characters, the text of a panel, Kirtley et al., 2018). Furthermore, a study by Foulsham and Cohn (2021) found that panels manipulated to show only the focal points of a panel still provided enough information for readers to understand the sequence. Back‐end processes relate to the construction of the overall situation model—the mental representation of the ongoing story shown across the individual panels. The information obtained will activate stored knowledge relating to the panel content. This stored knowledge, along with the incoming information, allows the reader to form expectations about what they will see next. Disruptions to these expectations will increase the viewing time on subsequent panels as viewers try to incorporate the unexpected information, while in sequences that progress as expected, less time is spent on the later panels (Foulsham et al., 2016). These expectations formed in the back‐end processes can also influence the front‐end processes in a feedback loop: that is, the direction of subsequent eye‐movements to upcoming regions that would confirm these predictions. Indeed, Foulsham et al. (2016) showed that eye‐movements become more directed to key locations, such as recurring characters, as the reader reads through the comic strip.

The semantic pathway of PINS similarly proposes that readers are making certain predictions about upcoming events in a narrative sequence, based on the content of the panels they have read so far. Again, coherent, predictable narratives are processed more quickly than those that contain a disruption (e.g., deletion or replacement of key panels), shown by larger N400 responses when unexpected semantic events occur (Cohn & Kutas, 2015; Cohn, Paczynski, Jackendoff, Holcomb, & Kuperberg, 2012). While panel content is dealt with by the semantic pathway, the narrative pathway is concerned with the sequential order of panels, and their roles in the narrative (Cohn, 2014). Readers are similarly sensitive to these aspects, showing high levels of agreement on the best sequence for panel groups (Cohn, 2014), and able to predict the type of panel that is likely to appear next in a sequence (Cohn & Kutas, 2015).

For both PINS and SPECT, the gathered information is used to update the ongoing situation model of the sequential narrative, which is held in working memory and elaborated on with each subsequent panel. Should the discontinuity between the stored model and upcoming information grow too great (e.g., the narrative shifts to a new scene or character, or a new segment is established), a new model will be created, and the old one stored in long term memory (LTM) (e.g., Cohn & Kutas, 2015).

Both PINS and SPECT provide strong frameworks from which subsequent research questions can be developed. One such question is the influence of peripheral information on the visual narrative reading process. That is, when readers are fixating on the current panel, are they also obtaining information from the upcoming panels, and what kind of information are they gathering? This has been a central question in the progress made in understanding the processes that underlie text reading and the development of computational models of this process (e.g., Murray, Fischer, & Tatler, 2013), but little work has been carried out on information processing in peripheral panels. While PINS and SPECT emphasize the importance of reader prediction, they focus only on prediction based on the content of the current fixation location. Indeed, several of the studies used a single panel presentation, so that readers never had multiple panels available to them at once (e.g., Experiment 1 from Cohn et al., 2014; Foulsham et al., 2016). However, Cohn (2020a) notes that, given the observed ordered progression of eye movements through panels, it is reasonable to consider a role for peripheral vision in guiding ongoing fixations.

Despite the lack of research on this question, there is evidence from exploratory studies that the next panel is processed to some extent before the reader directly fixates it. If text is placed at the top of the panel, or is close to the border with the preceding panel, the reader's first fixation is more likely to fall within the text region, compared to situations where the text is further from the preceding panel (Kirtley et al., 2018; Omori, Ishii, & Kurata, 2004). This suggests at least some structural encoding that is sufficient to identify text regions, if they are close to the current panel. Panels are more likely to be skipped (either not directly fixated or read out of sequence) when they do not contain text or contain an image that focuses on a background scene or object rather than a character (Kirtley et al., 2018). There is also a suggestion that information can be gained from panels beyond the neighboring one: while textless panels are more likely to be skipped, the probability of this is increased further if the panel following it contains text (Kirtley et al., 2018).

More specifically, Laubrock, Hohenstein, and Kümmerer (2018) corpus study showed evidence that readers are preprocessing both text and images in upcoming panels, as evidenced by short fixation durations on new panels following shorter saccades (when readers have not moved as far, and thus have had access to a more detailed preview of the upcoming location). There is also evidence of foveal load effects for text specifically, where less preview information is obtained when the currently fixated text region is more difficult (i.e., contains lower frequency words). Such findings indicate that both text and image components of upcoming panels are preprocessed to some extent when planning a saccade. However, it is not clear what the extent of the preview effect is: that is, from how many panels ahead of their current fixation location readers are able to draw and use information.

A powerful experimental tool for testing the spatial extent of extra‐foveal processing during reading is the moving‐window paradigm (McConkie & Rayner, 1975). This technique restricts the amount of peripheral information available to the reader by either removing or masking information beyond a certain point to the right or left of fixation. Readers accustomed to a left‐to‐right reading system are able to process information from 14−15 character spaces to the right of their current fixation location, and only 3−4 to the left (Rayner & McConkie, 1976; Rayner, Well, Pollatsek, & Bertera, 1982). When changes happen outside these boundaries, readers show very little awareness of the fact, and their reading speed is not strongly affected (Rayner et al., 1982). This technique has been used to demonstrate the spatial extent over which different types of information appear to be processed in reading. Structural information, such as word length and spacing, can be extracted around 14−15 characters to the right of central fixation (Rayner, 1986), whereas letter identity can only be extracted for the next nine characters to the right of fixation (Haikio et al., 2009). In the current study, we employed this moving window technique to consider the extent to which the absence of previews of upcoming panels influences measures of comic reading.

Similar questions have been debated and techniques employed in the scene viewing literature. Saida and Ikeda (1979) used the moving window paradigm in a scene viewing study, and found that the area from which information is gathered is much wider: around 50% of the image needed to be available to viewers in their periphery for their viewing behavior to remain unaffected. The information that is available is clearly used by the viewers to guide their upcoming saccades: when restricted by differently shaped moving windows, participants planned their fixations within the available areas, rather than making new fixations in regions they had no information for (Foulsham, Teszka, & Kingstone, 2011). Given the very different extents to which peripheral information is used when reading text versus viewing images, it is not clear how this information would be used in a situation where both text and image are present, such as comics.

### 1.1 Current study

We present two experiments with the aim of investigating how peripheral information affects reading in sequential narratives, composed of text and images. Both experiments used the moving window paradigm to determine whether reading was disrupted when peripheral information was removed, and the extent of this disruption, in two different versions of typical layouts. In Experiment 1, we focused on short, single‐line comic strips taken from newspaper comics. In Experiment 2, we used longer comic stories, with multiple lines per page. This allows us to explore the influence of peripheral visual information in a more complex arrangement. The present study is the first experimental investigation of peripheral influences in sequential narratives, and the findings obtained will provide necessary information for existing models of sequential visual narratives regarding the role of peripheral vision.

## 2 Experiment 1 method

### 2.1 Participants

Forty participants (4 males, *M*_age_ = 21.37, *SD* = 2.97) were recruited from the Psychology undergraduate population at the University of Aberdeen. Participants’ comic expertise was measured using the Visual Language Fluency Index (VLFI; Cohn, 2014a). The average score was 10.40 (*SD* = 5.69), indicating a low level of expertise in comics. They received course credits in return for their participation in the study. All participants had normal or corrected‐to‐normal vision. The study was approved by the local ethics committee (reference PEC/4029/2018/10) and was in accordance with The Code of Ethics of the World Medical Association (Declaration of Helsinki). Two participants were excluded from the study due to major calibration issues.

### 2.2 Stimuli and apparatus

#### 2.2.1 Comics

**Fig. 1:**
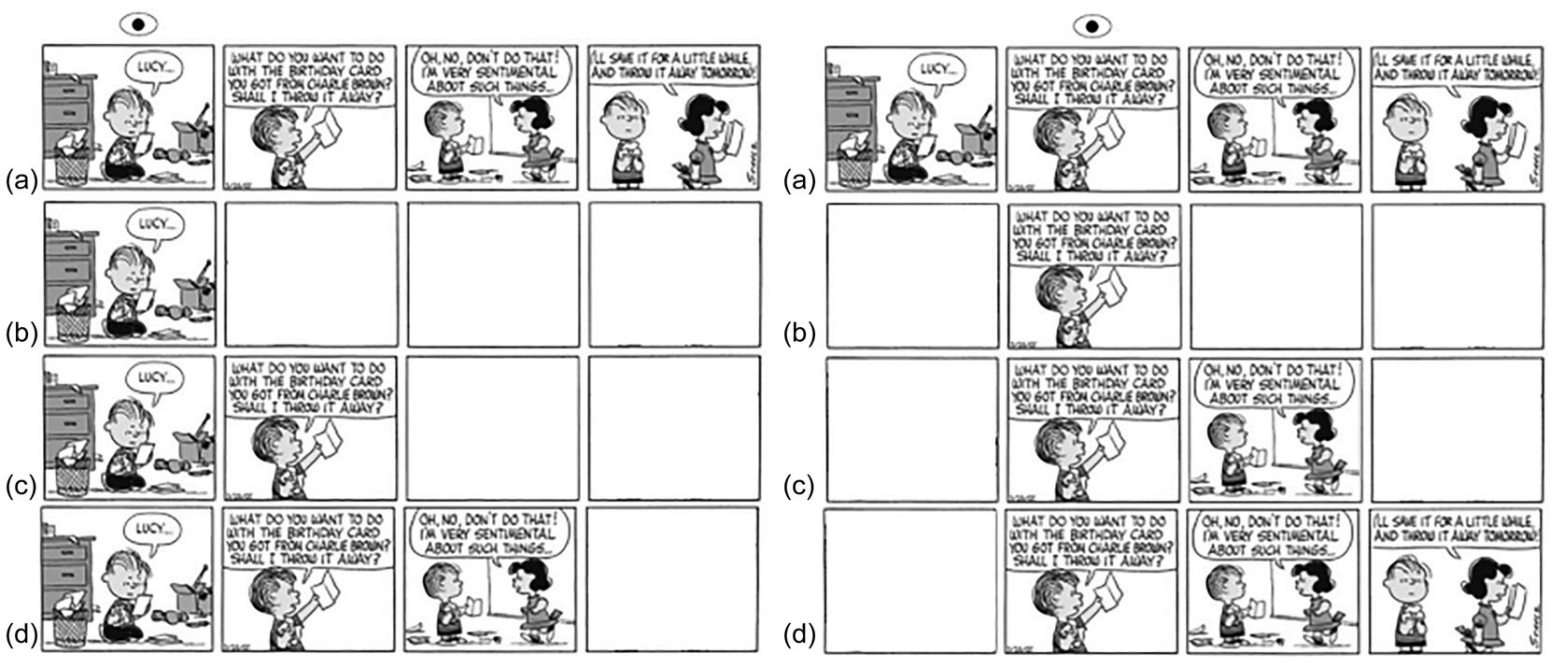
The four viewing conditions used in Experiment 1. *Notes*. Eye icon indicates the fixation position. Left: Reader views panel 1. Right: Reader views panel 2. The four conditions: A: *Control*; B: *n*; C: *n* + 1; D: *n* + 2.

Thirty‐two comic strips from the American daily strip series *Peanuts* (Schulz, 1950−2000) were chosen from the website of an independent syndicate GoComics. All of the chosen comics presented a complete short story in four identical square‐shaped panels, and were read following the standard Western left‐to‐right direction. An example of a strip can be seen in Fig. 1. Sixteen strips contained text in each panel (either speech or captions), and 16 strips were image only. Stimulus presentation and response collection was controlled by the Experiment Builder program from SR Research. The comics were displayed to the participants on the display monitor of the EyeLink 1000+, at a resolution of 1024 x 768, and with a refresh rate of 60 Hz. Participants viewed the screen from a distance of 72 cm, and the screen subtended 40.6° horizontally and 23.5° vertically.

#### 2.2.2 Eye‐tracking

Participants’ eye movements were monitored and recorded on the EyeLink 1000+ eye‐tracker, set to the participants’ dominant eye. A 9‐point calibration grid, followed by a 9‐point validation grid, was used to fit and test the spatial accuracy of the eye‐tracker at the start of the experiment. Eye dominance was determined using a variant of the Miles test (Miles, 1929). If the validation procedure showed a mean spatial accuracy worse than 0.5 degrees, or a maximum spatial accuracy worse than 1 degree, calibration and setup were repeated. Saccades were detected using the default cognitive setting for the SR Research algorithm.

#### 2.2.3 Expertise questionnaire

Participants’ familiarity with comics as a medium was assessed using the VLFI (Cohn, 2014a). This questionnaire asks participants to rate, on a scale of 1−7, the frequency with which they read and drew varieties of comics, both currently and during childhood (aged 16 or younger). They are also asked to rate their proficiency in drawing both currently and during childhood, and how fluent they consider themselves to be in comic reading. The VLFI score ranges from 1 to 52.5, with higher numbers indicating greater comic fluency.

### 2.3 Procedure

Participants were asked to read the comics at their own pace while their eye movements were tracked. Prior to the presentation of a strip, a fixation point appeared at the left of the screen (at xy coordinates 45, 512). When participants fixated this point, the study progressed to show the comic, and readers did not start the trial fixating in the center of the strip, but to the left of the first panel, requiring them to make a saccade into it. Strips were presented in the middle of the screen and appeared in one of four conditions. In the Control condition, the story was presented with no changes to panel visibility. In condition *n*, only the panel being fixated was visible to the participant. Surrounding panels (to both the left and right of the fixated panel) were blanked out, and would only show information when a fixation was made within the panel region. Once the reader left the current panel and moved to a new one, the old panel would become blank again (see Fig. 1, right‐hand side, below, for an example of how this appeared to participants in each condition). In the *n* + 1 condition, the fixated panel, and the panel to its right (i.e., the next panel in the reading sequence) were visible, while all other panels were blanked out. In the *n* + 2 condition, the fixated panel, and the two following panels were visible. The viewing conditions were counterbalanced across the six stories. The moving window was controlled using invisible boundaries between each panel, such that whole panels were masked or revealed depending on which panel was being viewed. This was not influenced by where fixations landed within the panel. Reading was self‐paced; participants pressed a key when they had finished reading one strip, to move on to the next. Fig. 1 displays examples of the four conditions.

The study typically took less than 30 min to complete. After reading the stories, participants were given the VLFI to complete on paper.

### 2.4 Analysis

To analyze the various possible influences on reading behaviors in comics, linear mixed models (LMMs) were run using the lme4 package (Bates et al., 2015) in the R statistical programming environment (R Core Team, 2024). To meet the assumptions of normal distribution required for LMMs, the dependent eye movement measures were adjusted using log transformations if required. *p*‐Values were generated using the lmerTest library (Kuznetsova et al., 2017). When an interaction was significant, we ran follow‐up models to explore it. Data plots were created using the ggplot2 package (Wickham, 2016).

The main concern of the experiment was how the viewing condition changed reading behavior. Measures of eye movement behavior examined included viewing time (the average total time spent on individual panels); the landing position of the first fixation into a new panel (on regions of text or image); the proportion of first‐pass panel skipping (where the panel is fixated after panels that come later in the sequence), and the proportion of regressions (the incidences of readers returning to panels they have previously fixated, or launching such saccades from panels) between the individual panels. These measures of comic reading allow us to examine both the time spent on key regions and disruptions to normal reading, shown in the Control condition (see Kirtley, Murray, Vaughan, & Tatler, 2023 for more details on these measures).

For each measure, we modeled the effect of the viewing condition, the participants’ expertise, the presence of text in the panels, and the interaction between text presence and viewing condition. Fixed effects were coded using simple coding. The participant and the comic strip were included as random effects. All model structures are provided in Appendix 1, and full model outputs in Appendix 2.

## 3 Experiment 1 results

### 3.1 Viewing time per panel

Previous work (Kirtley et al., 2018) has shown that the presence of text in a panel increases viewing time. We examined whether this would interact with the viewing condition in influencing the time spent viewing panels. Dwell time was log‐transformed to ensure the data were normally distributed.

Viewing condition had a significant influence on viewing time—readers took longer to view the panels when their vision was restricted to only the current (*n*) panel (*M* = 2632 ms, *SD* = 2558) compared to the control condition (*M* = 1912 ms, *SD* = 1538). No difference was found between the Control and *n* + 1 condition (*M* = 2120 ms, *SD* = 2441), or Control and *n* + 2 (*M* = 2068 ms, *SD* = 1853).

The presence of text was also a significant predictor of viewing time, such that panels containing text (*M* = 2738 ms, *SD* = 2105) were examined for longer than textless panels (*M* = 1629 ms, *SD* = 2055). The readers’ expertise had no influence on viewing time.

Table 1 summarizes the influence of these factors on the average total dwell time.

**Table 1:** Summary of factors influencing viewing time per panel

Factor	*β*	*SE*	*t*	*p*
Expertise	−0.002	0.004	−0.45	.657
Contains Text	0.135	0.013	−10.52	<.001
Control versus *n*	0.131	0.015	11.39	<.001
Control versus *n* + 1	0.018	0.011	1.55	.122
Control versus *n* + 2	0.012	0.015	1.07	.283
Text*Condition(*n*)	−0.046	0.011	−4.02	<.001
Text*Condition(*n* + 1)	−0.003	0.011	−0.24	.810
Text*Condition(*n* + 2)	0.001	0.011	0.053	.958

**Fig. 2:**
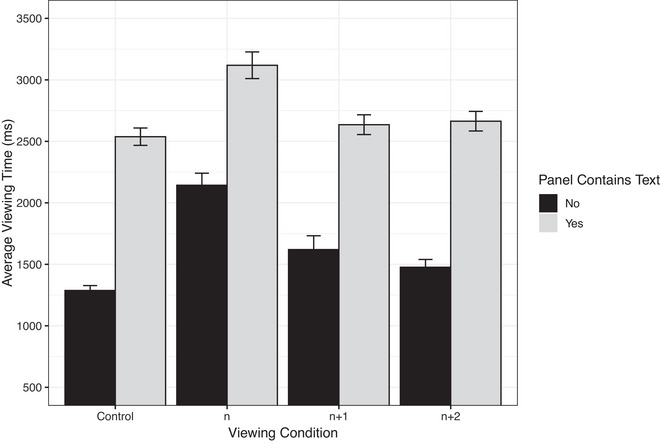
Viewing times per panel (with and without text) across the four viewing conditions. *Note*. Error bars represent one standard error.

Text presence and viewing condition showed a significant interaction, specifically for condition *n*, where increased time was spent on both textless and text‐containing panels when no preview was available. Fig. 2 shows this interaction.

This interaction was further explored by comparing time on panels with or without text across the four conditions, using Tukey pairwise comparisons. For condition *n*, with no preview available, both panels with text (*p* < .001) and without (*p* < .001) were viewed for longer than such panels in the control viewing condition. For conditions with reduced preview (*n* + 1 and *n* + 2), the time on panels with or without text did not significantly differ from the control condition (all *p*’s > .7).

### 3.2 Landing position

Readers tend to make their first fixation to the text of the upcoming panel, and evidence suggests that if text is somehow more visible to the reader while they are fixating on earlier panels, they are more likely to do this (Omori et al., 2004; Kirtley et al., 2018). We examined how the absence of visual preview affects the readers’ tendency to make the initial fixation to the text of the upcoming panel. While previous work has found that the location of the text in the panel can increase the likelihood of first fixating the text, all examples of the Peanuts comics presented the text at the top of the panel. As a result, only the viewing condition and readers’ expertise were included as factors. For this analysis, only panels that contained both text and image were considered.

**Fig. 3:**
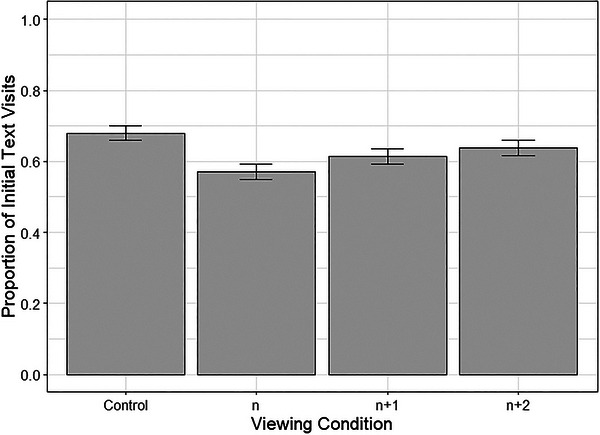
Average proportion of first fixations to the text region across the four viewing conditions. *Note*. Error bars represent one standard error of the mean.

In the control viewing condition, 67.9% of first fixations were made to the text region of a panel. Incidences of this were significantly reduced when no preview was available, to 57.1%, *β* = −0.532, *SE* = 0.136, *z* = −3.92, *p* < .001. When a one‐panel preview was available, 61.4% of first fixations were made to the text, which was again found to be significantly lower than this behavior in the control condition, *β* = −0.321, *SE* = 0.138, *z* = −2.33, *p* = .020. While first text fixations in the *n* + 2 preview condition were also reduced to 63.8%, the difference from the control condition was not significant, *β* = −0.185, *SE* = 0.138, *z* = −1.34, *p* = .180. Expertise had no influence on landing position, *β* = −0.013, *SE* = 0.022, *z* = −0.58, *p* = .563. Fig. 3 shows the average proportion of first fixations to the text across the four conditions.

### 3.3 First pass skipping of panels

Earlier work has shown that readers’ preference for text means they are less likely to skip over panels that contain text (Kirtley et al., 2018). Here, we examined the proportion of first pass skips, where the reader does not visit the panel until after viewing panels that appear later in the sequence, and how this was influenced by viewing condition, text presence, and expertise. In this study, first pass skips were found to be rare, with an average of 2% across all conditions. Examining the factors influencing this behavior showed no significant effects of expertise or text presence, and no interaction between condition and text presence. Table 2 summarizes the influences of these factors on first‐past skipping behavior.

**Table 2:** Summary of factors influencing first‐past skipping behavior

Factor	*β*	*SE*	*z*	*p*
Control versus *n*	0.606	0.306	1.98	.048
Control versus *n* + 1	0.409	0.316	1.30	.195
Control versus *n* + 2	0.006	0.340	0.016	.987
Contains Text	0.169	0.108	1.56	.120
Expertise	0.012	0.049	0.240	.810

For the viewing condition, more skips occurred in condition *n* (*M* = 0.036, *SD* = 0.186) compared to control (*M* = 0.021, *SD* = 0.143). The proportion of skipping in condition *n* + 1 (*M* = 0.030, *SD* = 0.171) or condition *n* + 2 (*M* = 0.021, *SD* = 0.144) did not differ significantly from the control condition.

### 3.4 Regressions to earlier panels

While there is some evidence to suggest that comic readers make regressions between panels when there is confusion over meaning or reading order (Kirtley et al., 2023), regressions also seem to be a common part of the reading process for this medium, with more incidences seen than in standard text reading (e.g., Foulsham et al., 2016, Kirtley et al., 2018). Here, we examined how the viewing condition might affect this behavior. In this study, of saccades that were made between panels (starting in one panel and ending in another), the majority were movements forward (78.1%), while 21.9% were regressions, taking the readers back to an earlier panel.

Regression behaviors were affected by the viewing condition, with significantly fewer regressions taking place in conditions when any limit was placed on the preview. For the Control condition, 31.4% of between panel saccades were regressions, while this was reduced to 14.3% for condition *n*, 19.4% for condition *n* + 1, and 18.1% for condition *n* + 2.

Neither participants’ expertise nor the presence of text in the panel that the regression entered was a significant influence, and no significant interaction was observed between text presence and viewing condition.

Table 3 summarizes the results from this analysis.

**Table 3:** Summary of factors influencing proportion of regressions to earlier panels

Factor	*β*	*SE*	*z*	*p*
Expertise	0.016	0.019	0.86	.387
Contains Text	0.043	0.057	0.77	.444
Control versus *n*	−1.07	0.105	−10.15	<.001
Control versus *n* + 1	−0.785	0.090	−8.72	<.001
Control versus *n* + 2	−0.806	0.093	−8.68	<.001
Text*Condition(*n*)	0.159	0.105	1.52	.129
Text*Condition(*n* + 1)	0.130	0.090	1.45	.146
Text*Condition(*n* + 2)	0.060	0.092	0.651	.515

## 4 Experiment 1 discussion

Experiment 1 used the moving window paradigm to restrict the amount of information available to the reader beyond their immediate fixation, in short, simple comic strips. Completely removing any peripheral information to the right and left of the currently fixated panel slowed reading time significantly. Other behaviors were also affected: while readers in the control condition typically made their first fixation to a panel's text, if the preview was entirely removed or restricted to one panel, this was reduced. First pass skips showed a small effect of preview manipulations, and regressions to earlier panels were also reduced when the preview was restricted. Readers’ viewing behavior, therefore, is affected by the availability of information in upcoming panels, and they use this to guide their fixations into these panels.

While Experiment 1 focused on short comic strips, many comics are longer, with sequences across several lines within one page. Previous work has shown that some reading behaviors (e.g., skipping) are more prevalent in these more complex designs (e.g., Kirtley et al., 2018). This may reflect differences in the amount of information readers are able to extract from upcoming panels in these two comic types. In Experiment 2, we expand upon the findings of Experiment 1 by manipulating the available preview for longer, multiline comic stories. In this way, we can examine whether comic structure influences the use of preview and upcoming information. Readers may show that they require a preview of several panels ahead for more complex layouts, for reading behavior to remain unchanged. Alternatively, as multiline comics tend to be more reduced in the number of panels per line (typically 2−3, rather than 4), a single‐panel preview may still be suitable for readers to maintain their usual reading behavior.

## 5 Experiment 2 method

### 5.1 Participants

Thirty participants (5 males, 1 nonbinary, *M*_age_ = 20.37, *SD* = 3.33) were recruited from the Psychology undergraduate population at the University of Aberdeen. None of these individuals had participated in Experiment 1. The average VLFI score of the sample was 10.09 (*SD* = 6.85). They received course credits in return for their participation in the study. All participants had normal or corrected‐to‐normal vision. The study was approved by the local ethics committee (reference PEC/3626/2017/4) and was in accordance with The Code of Ethics of the World Medical Association (Declaration of Helsinki).

### 5.2 Stimuli and apparatus

#### 5.2.1 Comics

**Fig. 4:**
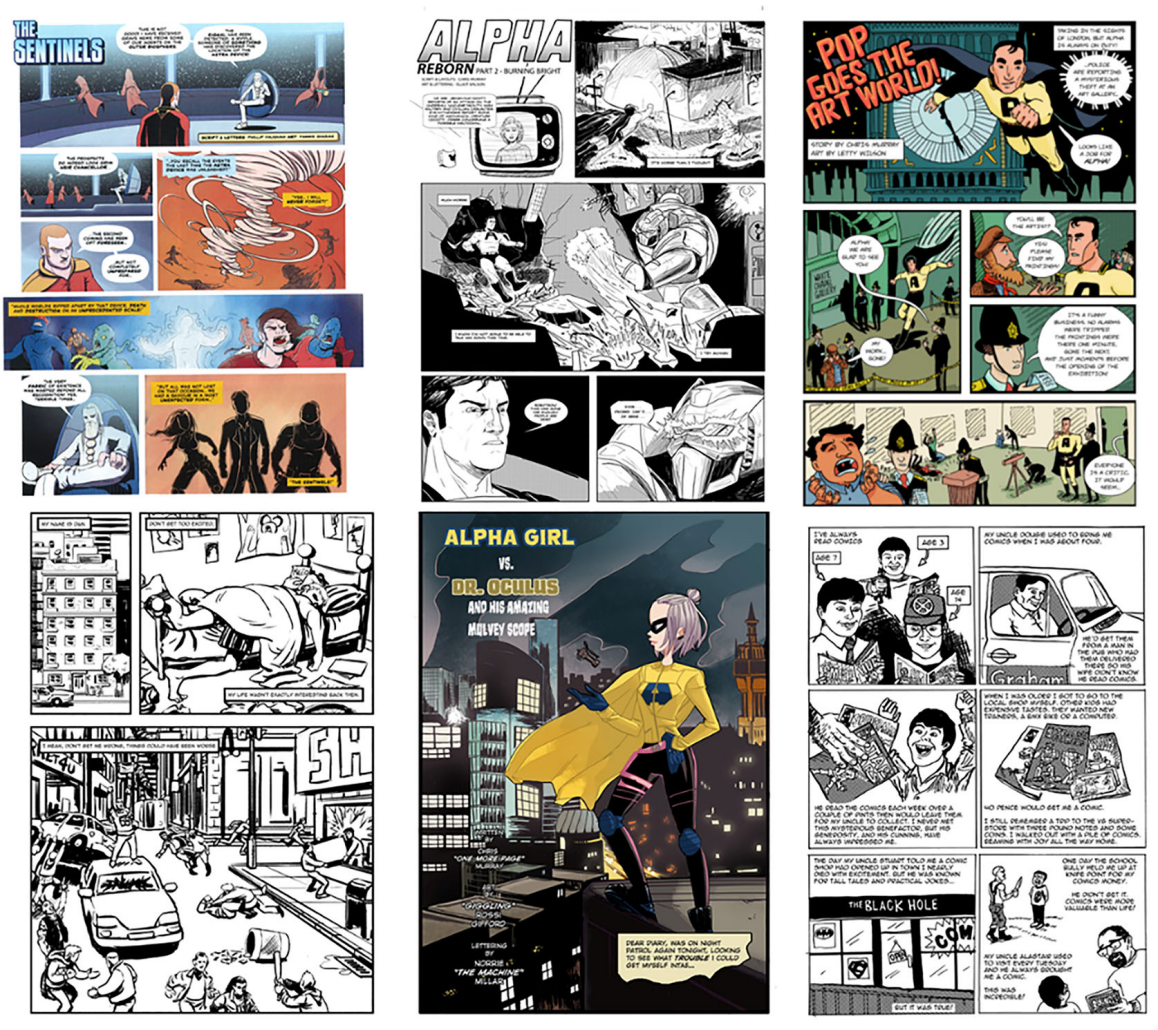
First pages of the six multipage comics used in this study, demonstrating the range of styles and content.

Local comic artists and writers created six comics for the purposes of several studies run in our lab. These comics portrayed a range of art styles, genres, and use of color versus black and white. Comics ranged from 3 to 6 pages in length, and sample pages from each of the six comics are shown in Fig. 4. All comics followed the left‐to‐right reading direction. See Kirtley et al. (2023) for more details on the comics used.

Stimulus presentation, eye‐tracking method, and expertise questionnaire were identical to Experiment 1A.

### 5.3 Procedure

**Fig. 5:**
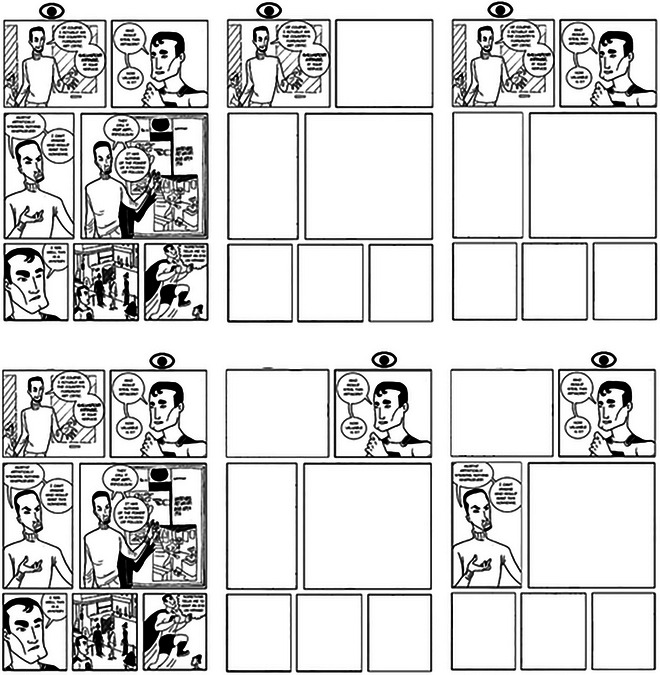
Example of the three viewing conditions, with eye icon indicating currently fixated panel. Left‐Right: Control, *n*, and *n* + 1. *Notes*. Upper image: Reader is viewing Panel 1. Lower image: Reader is viewing Panel 2.

Participants were asked to read the comics while their eye movements were tracked. Stories were presented as two pages per screen, at a size of 1024 x 768 pixels. Reading was self‐paced: once readers had finished reading the two pages, they pressed a key to move to the next set of pages. The stories appeared in one of three conditions (two stories per condition for each participant): Control, *n*, and *n* + 1, with the order of viewing randomized for each participant, and all pages of an individual story presented in the same condition. The preview restrictions of these conditions were identical to those used in Experiment 1. As the comics in Experiment 2 extended across multiple lines, in condition *n* + 1, when readers reached the end of a line, they received a preview of the next panel in reading order (i.e., the first panel of the next line). Viewing conditions were counterbalanced across the six stories. Fig. 5 displays examples of the three conditions.

Reading was self‐paced, and the study typically took less than 30 min to complete. After reading the stories, participants were given the VLFI to complete on a laptop computer.

### 5.4 Analysis

Analysis of the data was similar to that used in Experiment 1. Again, the measures used were viewing time per panel, landing position, proportion of first‐pass panel skipping, and the proportion of regressions. Viewing condition, text presence, and expertise were included as fixed effects in all models, with text location and word number per panel used as additional fixed effects in certain models, specified in the Results section. All fixed effects were entered into the models using simple coding. Interactions between condition and text presence were investigated for each model using simple effects. Participant and comic item were included as random effects. All model structures are provided in Appendix 1 and full model outputs are included in Appendix 3.

## 6 Experiment 2 results

### 6.1 Viewing time per panel

As in Experiment 1, the presence of text in panels and the participants’ expertise score were included as factors in the model of viewing time, alongside the factor of reading condition. Viewing time was log‐transformed to ensure normal distribution.

**Fig. 6:**
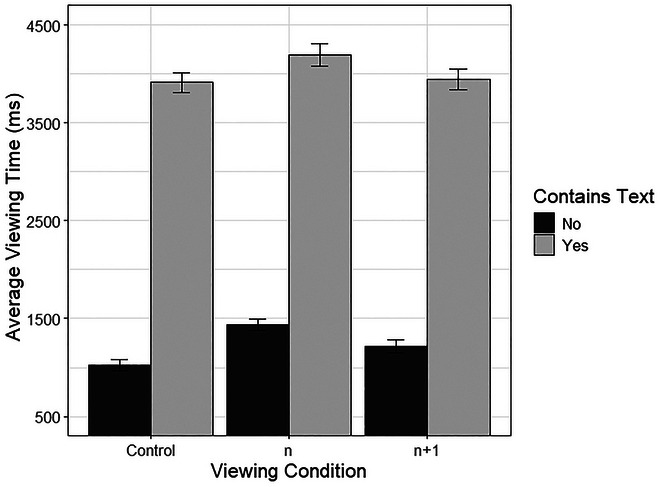
Viewing times per panel (with and without text) across the three viewing conditions. *Note*. Error bars represent one standard error.

Readers spent longer on panels when all preview information was removed (*M* = 3585 ms, *SD* = 3764) than when all the panels were available to them (*M* = 3324 ms, *SD* = 3473). Viewing time was also significantly increased in condition *n* + 1 (*M* = 3363 ms, *SD* = 3554) compared to the control condition. The presence of text also increased time spent on panels (*M* = 4053 ms, *SD* = 3792) compared to textless panels (*M* = 1071 ms, *SD* = 746). A general effect of expertise was found, with more expert readers spending longer on panels regardless of viewing condition. Significant interactions between text presence and viewing condition were found in both restricted viewing conditions. This was further explored using Tukey‐corrected pairwise‐comparisons across textless panels and those containing text separately. As in Experiment 1, for condition *n*, panels with text (*p* = .001) and without (*p* < .001) were viewed significantly longer than such panels viewed in the control condition. A different pattern emerged for condition *n* + 1, however. Here, for panels containing text, there was no significant difference in viewing time compared to the Control condition, *p* = .970. Only for textless panels viewed in condition *n* + 1 was there a significant increase in viewing time relative to control, *p* < .001. Fig. 6 illustrates this interaction.

Table 4 summarizes the influence of the factors on the average total dwell time.

**Table 4:** Summary of factors influencing viewing time per panel

Factor	*β*	*SE*	*t*	*p*
Expertise	0.007	0.003	2.23	.03
Contains Text	0.210	0.006	36.74	<.001
Control versus *n*	0.130	0.014	9.43	<.001
Control versus *n* + 1	0.031	0.014	2.28	.02
Text*Condition(*n*)	−0.076	0.014	−5.55	<.001
Text*Condition(*n* + 1)	−0.039	0.014	−2.83	.005

### 6.2 Landing position

Reader's tendency to first fixate the text or image regions of upcoming panels was again considered. For this analysis, only panels that contained both text and image were included (88% of all panels). As there was more variance in where text could appear in a panel (top or bottom of the space), this was included as a predictive factor in the model.

**Fig. 7:**
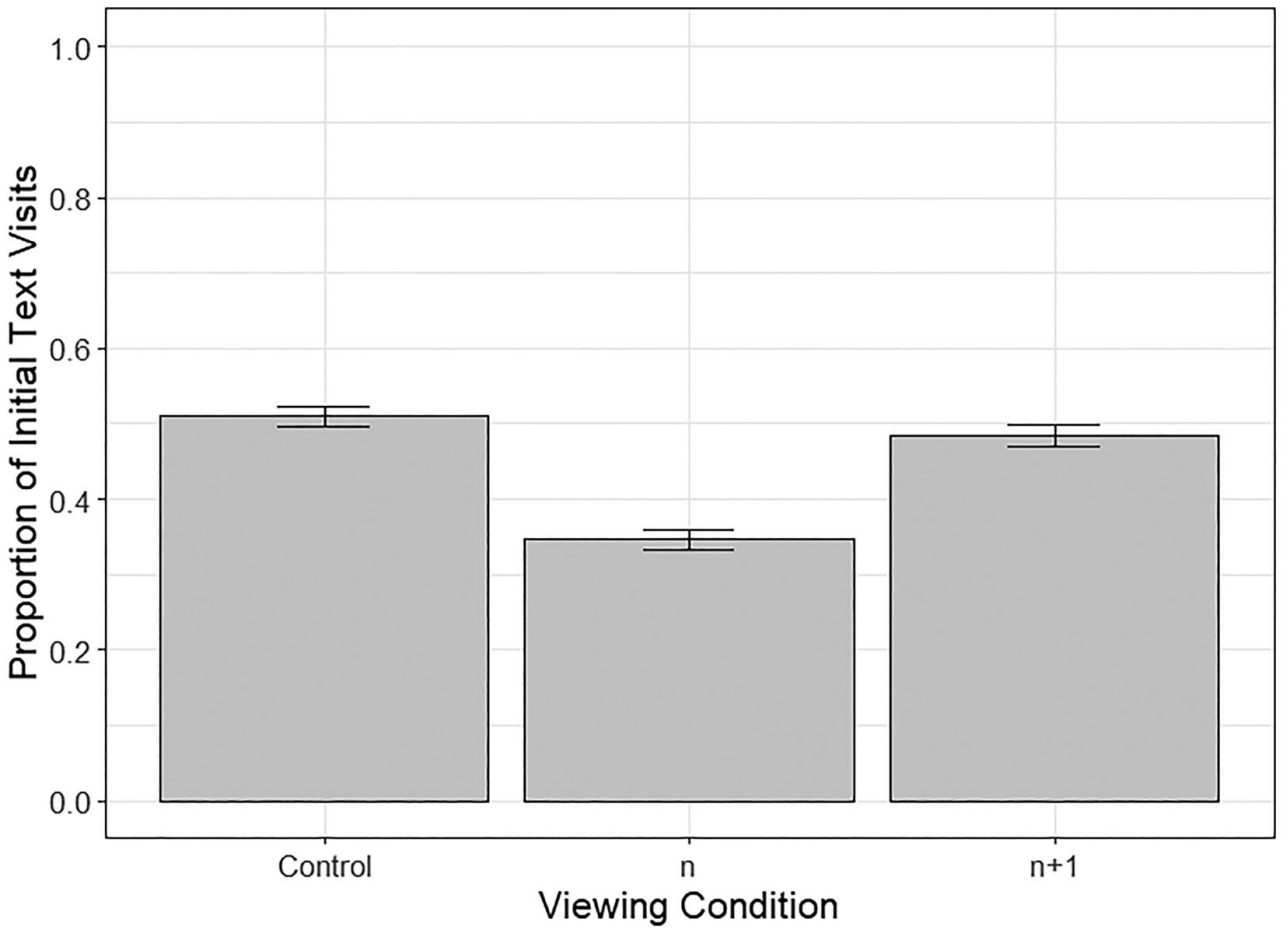
Average proportion of first fixations to the text regions. *Note*. Error bars represent one standard error.

Text location affected the first fixation, *β* = −0.756, *SE* = 0.075, *z* = −10.13, *p* < .001, so that readers were more likely to make the first fixation to the text when it was presented at the top of the panel. Furthermore, viewing condition was found to influence landing location between the Control and *n* viewing conditions. Without a preview of the upcoming panel, 34.6% of first fixations to a panel containing text and image were to the text, while significantly more first fixations were made to text in the control condition (51%), *β* = −0.804, *SE* = 0.092, *z* = −8.77, *p* < .001. Control *n* + 1 conditions did not differ in the landing location of the first fixation in a panel, with 48.4% of first fixations to the text, *β* = −0.100, *SE* = 0.087, *z* = −1.146, *p* = .252. However, expertise had no influence on landing position, *β* = −0.011, *SE* = 0.012, *z* = −0.958, *p* = .338, and no interactions between text location and viewing condition were found. Fig. 7 shows the average proportion of initial visits to text regions across the three conditions.

### 6.3 First pass skipping of panels

Proportions of skipping were higher in Experiment 2 than 1, with 9.10% of panels being skipped in the first pass reading. The model included the viewing condition, as well as the presence of text in panels, and whether panels had a neighboring panel directly to their right (i.e., on the same line as the panel).

Overall, readers were less likely to skip panels that contained text (0.098, *SD* = 0.298) compared to panels without text (0.127, *SD* = 0.333). Significant differences also emerged when comparing the experimental viewing conditions to Control; the average proportion of first pass skips in the Control condition was 0.146 (*SD* = 0.353), but only 0.087 (*SD* = 0.281) in the *n* viewing condition and 0.061 (*SD* = 0.239) in the *n +*1 condition. Having a neighboring panel on the same line as the current panel was not a significant influence on the likelihood of skipping (same line: 0.111, *SD* = 0.314; lower line: 0.098, *SD* = 0.298), and participants’ expertise did not influence their likelihood of skipping. As skipping behavior was more likely for these comics, the interaction between condition and text presence was also explored. A significant interaction was found between the presence of text in the immediately following panel and the viewing condition: the full model is summarized in Table 5.

**Table 5:** Summary of factors influencing first pass skipping behavior

Factor	*β*	*SE*	*z*	*p*
Control versus *n*	−0.719	0.146	−4.92	<.001
Control versus *n* + 1	−1.20	0.169	−7.11	<.001
Contains Text	0.361	0.10	3.72	<.001
Expertise	−0.013	0.022	−0.60	.547
Neighboring Panel	0.170	0.111	1.53	.125
Text*Condition(*n*)	−0.106	0.145	−0.73	.464
Text*Condition(*n* + 1)	−0.500	0.168	−2.98	.003

**Fig. 8:**
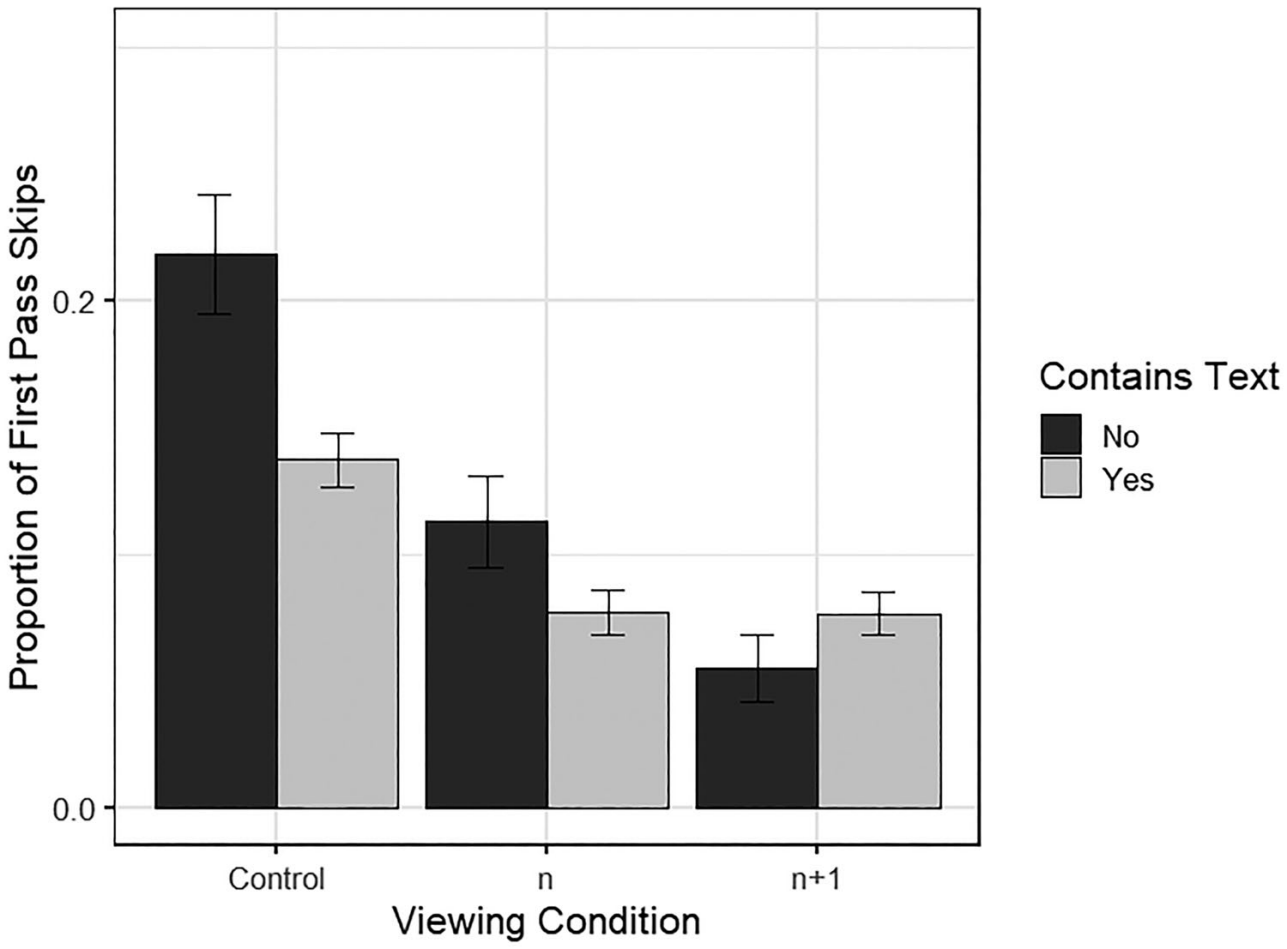
Proportion of first pass skips occurring according to the presence of text and viewing condition. *Note*. Error bars represent one standard error.

Further investigations of the interactions showed that readers were significantly more likely to skip panels that contained no text when they viewed them in the control viewing condition, *t*(428) = 3.37, *p* < .001. However, the presence of text had no influence on skipping panels when the preview was removed or reduced. Fig. 8 illustrates this finding.

### 6.4 Regressions to earlier panels

Significant differences in the number of regressive saccades did emerge between the three conditions. In the Control condition, when the preview was unrestricted, 27% of between‐panel saccades were to earlier panels. In the *n* condition, this was reduced to 18%, and in *n* + 1, to 18.1%. If a panel contained text, this increased the proportion of regressions back to the region. An interaction between text presence and viewing condition was also found for condition *n* + 1. Significantly more regressions occurred back to panels containing text in condition *n* + 1, *p* = .021. Expertise had no influence on this behavior. Table 6 summarizes the effects of condition on regression behaviors.

**Table 6:** Summary of factors influencing proportion of regressions to earlier panels

Factor	*β*	*SE*	*z*	*p*
Expertise	0.001	0.009	0.16	.873
Contains Text	−0.099	0.046	−2.15	.031
Control versus *n*	−0.691	0.104	−6.67	<.001
Control versus *n* + 1	−0.664	0.094	−7.05	<.001
Text*Condition(*n*)	−0.212	0.104	−2.04	.041
Text*Condition(*n* + 1)	−0.115	0.095	−1.22	.223

## 7 Experiment 2 discussion

Experiment 2 used the moving window paradigm to restrict the amount of information available to the reader beyond the immediately fixated panel for multiline comics. Like Experiment 1, it was found that the complete removal of upcoming panels slowed reading speed and affected other aspects of reading behavior, such as skipping, regressions, and first fixation locations—suggesting that information from the next panel is processed before it is directly fixated and contributes to reading behavior. In contrast to Experiment 1, a reduced amount of preview was associated with more varied patterns of behavior, suggesting that the processing of peripheral information differs when exploring more complex, multiline comics.

## 8 General discussion

Experiments 1 and 2 both showed the strongest influences on reading behavior when all preview was removed, resulting in longer viewing times per panel, reduced first fixations to text, reduced regressions to previous panels, and, in the case of longer, multiline comics, reductions in skipping of panels. Overall, our findings provide further evidence against the idea of comic reading as an erratic process (e.g., Groensteen, 2007), in which readers are moving between many different possible reading approaches (e.g., Hatfield, 2009).

Instead, we find that visual narratives are read in an ordered fashion, with readers showing sensitivity to disruptions, such as preview removal. Below, we compare the findings in these two studies and consider how this relates to previous work in this area.

### 8.1 No preview

Both Experiments 1 and 2 showed that participants’ time viewing a panel was most increased when they were faced with no preview (condition *n*). This suggests that important processing of the next panel takes place prior to the first direct fixation to this area. Longer fixation times within a panel, when there has been no preview, are likely to reflect the time needed to acquire the information they would usually have gained from a peripheral preview of the panel. Previous work by Foulsham et al. (2016) has found that the time spent viewing each panel will usually vary over a short narrative sequence, with more time spent on initial panels and less on later panels. This has been suggested to indicate initial construction of the situation model (Cohn, 2020b), and then easier addition of predictable information from the subsequent panels. In the current study, while readers are likely still making predictions about the next panels even without visual information in the periphery, the presence of the visual information, even as lower‐quality peripheral information, is still crucial in updating the ongoing model.

Another (not mutually exclusive) explanation for the increased viewing time comes from Foulsham et al. (2011), who found participants were unwilling to make saccades into a blank area. Previous work specifically on comics has also shown that readers are able to plan their saccades to areas of text in the next panel and use this to plan their saccades into the new region (Kirtley et al., 2018). This can be compared to Foulsham et al. (2016) suggestion that increased viewing times in their Experiment 1 might also have been due to readers realizing they would be unable to return to viewed panels, and thus they were taking the opportunity to understand the initial panels while they could, before moving on. In the current study, the restriction is not so great—readers are able to regress back to blank panels, although, as noted, they may be less willing. However, this restriction may still have had some role in readers giving each panel more time. Thus, the findings in the present study may reflect participants’ hesitancy before saccading to the next panel due to the uncertainty introduced by removing any indication of where important information will be found in the upcoming panel.

Both experiments found similar effects on landing position. In other studies of multimedia stimuli, readers have been shown to prioritize the text region. The typical pattern, seen when viewing single‐panel cartoons (Carroll et al., 1992), magazine adverts (Rayner, Sereno, & Raney, 1996), and photographs of real‐world scenes with text in the environment (Wang and Pomplun, 2012) is for a brief first fixation to the image, followed by a saccade to the text, where the reader remains for longer. However, in sequential comics, text is often the very first region to be fixated (Kirtley et al., 2018, Omori et al., 2004), since the reader typically has a preview of the upcoming information and can, therefore, plan their saccade to go directly to the text. Rayner et al. (1996) suggest that our experience with reading may lead us to expect text to be the most informationally dense section in a combination of words and images, and so prioritize it over the image content. (It should be noted that this text dominance in information content is not necessarily the case for all panels—see McCloud's (1993, 2006) examples of six text‐image combinations, where the division of information between text and image can vary widely. However, given that our participants were not particularly familiar comic readers, it seems safe to assume a general presupposition that text will always be the more informative part of the next panel.)

Even without a preview of upcoming panels, first fixations to text regions are still more common than first fixations to image regions in condition *n* (65% in Experiment 1, and 56% in Experiment 2). This suggests that there is a certain amount of expectation, or perhaps prior experience, influencing the planning of saccades into a new panel. The slightly higher level of first text fixations in Experiment 1 may be due to readers learning that text in the comic strips was always presented at the top of the panel, allowing them to anticipate this as a good starting location, even without a preview. While it is a relatively high proportion, first fixations of text regions in condition *n* were still significantly lower than in the control condition, illustrating that this is a purposeful behavior, based on preprocessing of the panel, rather than a default look to where text is expected to be. Thus, while readers can anticipate likely text locations, the preview information significantly increases the accuracy of this behavior.

The tendency to skip panels in first pass reading showed a difference between the two conditions. For Experiment 1, with no preview, skipping actually increased slightly. This seems to be due to readers’ behavior in the first few trials of condition *n*, which show significantly higher proportions of skipping than the other conditions (see Supplementary Materials), suggesting this is an influence of the paradigm. Readers may be initially uncertain at seeing blank spaces instead of filled panels—or be attempting to get a less restricted view of the comic and so saccade more erratically into the panels at first.

In contrast, for the multiline comics in Experiment 2, skipping was decreased in condition *n*. As in previous work (Kirtley et al., 2018), we found that skipping occurred more frequently for textless panels in Experiment 2. This might be either because readers think these will be less informative, or, in normal reading, they have already completed much of the textless panel processing while fixating the previous panel. With no preview, the reader has no information about the content of the next panel, and no chance for prefixation processing, and so there is not enough reliable information to inform the skip. Taken together, these findings add further support to the role of panel *n* + 1 in comic reading—an important source of information, assisting the reader in planning their upcoming saccades and fixations as well as contributing to the ongoing construction of the narrative situation model.

Regressions were reduced by the removal of any preview for the upcoming panel in both experiments. This may suggest that, without a preview, readers are processing each panel more completely (as seen in the longer viewing times per panel for conditions with no preview) and, therefore, having less need to return to earlier panels for reinspection. However, regressions were still reduced in the conditions with limited preview when viewing time per panel is no different from control, which argues against this suggestion. Another potential explanation for reduced regressions is one already suggested—simply that, due to the fact that the perceptual window masks panels behind the reader's current position, and readers are less willing to launch saccades into blank areas (Foulsham et al., 2011).

Experiment 2 showed a small variation on this effect compared to Experiment 1, with the presence of text in a panel increasing the likelihood of readers returning to said panel in Experiment 2 but not in Experiment 1. This is likely due to the longer, more complex story lines used in this version, leading to readers choosing to return to panels containing text to check details more frequently in the absence of continually present information in their periphery.

### 8.2 Restricted preview

While the effect of removing any preview on reading behavior was similar in Experiments 1 and 2, more variation between Experiments 1 and 2 was seen in the restricted preview conditions, when readers had some peripheral information available to them. Here, we specifically compare the *n* + 1 conditions, as only Experiment 1 included *n* + 2, and this condition showed no significant differences from the control version in any measures. The most striking difference between the two experiments is for viewing time and skipping behaviors. In Experiment 1, a single panel's worth of preview was enough for reading speed to return to normal, suggesting that, for these shorter comics, while readers do process some information in the peripheral, upcoming panels, this is limited to only the next panel in sequence. Similarly, skipping behavior was no different from the control condition, and skipping in general was much lower, both compared to Experiment 2 and previous work by Kirtley et al. (2018, 2023). This reduction is likely due to the shorter comic strips used. In longer stories, readers may be more inclined to consider some panels (particularly those with no text) as less important, or informationally dense, and, therefore, “safe” to skip, at least in the first pass. In contrast, for stories with only four panels, every panel may be seen as potentially important to the story, so that readers will tend to fixate each one. Alternatively, four panels are not a long strip, so readers may see no point in skipping anything. This general pattern of infrequent skipping for shorter strips is also seen in Foulsham et al. (2016), where for six‐panel strips, skipping behavior was reported as “very rare” (Foulsham et al., 2016, pg. 573).

For Experiment 2, the restricted preview condition still had an influence, reducing the amount of skipping behavior observed, even though, in this condition, the reader can determine the content of the next panel in peripheral vision. However, deciding to skip panel *n* + 1 is only one component of the decision to skip a panel—the second is where the next fixation will land, if not in the next panel in sequence. Kirtley et al. (2018) found that when panel *n* + 1 is skipped, the landing point is most commonly in panel *n* + 2, and furthermore, that the content of panel *n* + 2 also influences skipping. When panel *n* + 2 contains text and follows a textless panel (*n* + 1), skipping rates were at their highest. Readers may, therefore, use text presence to judge not only the potential low informativeness of the panel they skip, but also how much information they gain from the panel they instead move to. The present study, therefore, suggests that without information about *n* + 2—because preview has been restricted, readers do not choose to skip over upcoming panels.

Choosing to skip, or not, in this condition may also be responsible for the observed pattern of viewing time behavior, where textless panels in this condition were actually viewed for longer than panels containing text. We suggest that, on the rare occasions that skips do occur in this condition, readers do this without enough information, and so are more likely to discover that they have skipped something important, or that they cannot easily understand the panel they have skipped into. They then must return to the skipped panel, and process it in more detail, increasing the time spent viewing it, and leading to a disproportionate increase in time spent on textless panels specifically.

First fixations to text regions also showed more variation between Experiments 1 and 2. In Experiment 1, perhaps unexpectedly, first fixations to text were still significantly reduced in condition *n* + 1. This, a single‐panel preview, increased the proportion of fixations to text regions, compared to having no preview at all, but did not to the extent seen in the normal viewing condition. In contrast, in Experiment 2, the proportion of first fixations to text regions returned to normal with restricted preview; thus, a preview of only one panel was sufficient to return this aspect of reading behavior to that seen in normal, unrestricted comic reading.

The findings from Experiment 1 for where the first saccade landed suggest that there was also some information provided by panel *n* + 2, such that removing this affected the choice of first fixation location in panel *n* + 1. Alternatively, this may be due to the moving window manipulation itself: readers anticipate the reveal of *n* + 2 once they move into *n* + 1, and so saccade to a location in *n* + 1 which would provide a good launching point into *n +*2, in case it contains more information than *n* + 1. Meanwhile, the multiline comics in Experiment 2 may account for the lack of effect from restricting preview: usually, there are only two to three panels per line, so a preview of the next panel is all that is needed, and the reader is not trying to, or is unable to obtain the same information from the next panel in sequence, as it is on the line below. This does raise the question of whether readers are able to obtain any preview from panels that are directly below the currently fixated location, via a vertical preview. While the current study cannot provide an answer to this, it is an interesting point for future research.

Finally, both Experiments 1 and 2 show reduced levels of regression when the preview is restricted compared to that seen in normal, unrestricted reading. Again, a potential explanation is simply that readers are unwilling to saccade back into a blank area, such as the previous panels, which remain masked.

### 8.3 Peripheral information and models of sequential narratives

Together, these studies indicate the importance of peripheral information when reading comics, and suggest ways in which readers may be using the information in upcoming panels. From the first fixation location and skipping results, there is evidence that readers can identify the presence and location of text in the next panel, using this to plan their interpanel saccades. Furthermore, the extent to which this is done may depend on the complexity of the comic being read. Both SPECT and PINS (Cohn, 2020b) consider influences on where readers choose to fixate when reading a visual narrative, using panels as the basic visual unit of a comic. However, they do not differentiate between the panel the reader is currently fixating, and the panels in peripheral vision that have yet to be directly fixated (if they are available). Our findings speak particularly to SPECT's (Loschky et al., 2020) suggestion that back‐end processes (predictions and expectations) can influence the front‐end processes (eye movements). Removing this peripheral information source changes how fixations are directed, as seen particularly in the effects on landing position and skipping. Given the findings of the current study, we suggest that these models could be adjusted to differentiate between information gained from direct fixation and information from the next unit of the comic, since its removal affects the normal reading behavior of the viewer.

### 8.4 Future work and conclusions

The current study examines the extent to which readers can obtain peripheral information when reading comics. However, there are two further routes of enquiry, which can build on these findings. The first concerns the nature of the information that is being obtained from the preview. While we find readers are sensitive to preview removal from two panels ahead, it is important to determine exactly what they are accessing, whether locations of necessary information, or aspects of the meaning of the content. Similarly, in the current study, we focused on the influence of panel content, relating to SPECT (Loschky et al., 2020) and the semantic pathway of PINS (Cohn, 2020b). It is also important to explore what narrative information readers might be able to obtain from panel *n* + 1 and beyond, to determine whether the two streams of information are obtained from similar distances. The second line of enquiry relates to the specifics of the perceptual window paradigm. As mentioned above, the current study focuses only on horizontal preview, while multiline comics, such as in Experiment 2, also offer the potential for vertical preview, with readers potentially able to gain information from the panels on the line below, further ahead in the story. Finally, the stimuli used in both studies were Western comics, read in a left‐to‐right direction, as was the direction of the perceptual window. However, manga is typically read right‐to‐left, and, therefore, the results might differ if they were read with the rightward window used here.

In conclusion, we suggest that, as in scene viewing and text reading, peripheral information plays a key role when reading comics. Future work will allow us to further establish how similar this role is for sequential narratives, and where they exhibit their own unique rules, as a unique form of communication.

## Funding information

The current work was funded by the Economic and Social Research Council (ESRC; reference ES/M007081/1).

## Conflicts of interest

The authors confirm they have no conflicts of interest to declare.

## Ethics approval statement

The studies reported were approved by the University of Aberdeen ethics committee (Experiment 1 reference PEC/4029/2018/10; Experiment 2: reference PEC/3626/2017/4) and conducted in accordance with The Code of Ethics of the World Medical Association (Declaration of Helsinki).

This article includes a single reproduced *Peanuts* comic strip for the purpose of scholarly analysis and illustration of research methodology. The use is noncommercial and integral to the academic discussion. This limited use does not substitute for or compete with the original work, and it does not affect the market for *Peanuts* comics. The reproduction is, therefore, justified under the fair use doctrine (17 U.S.C. § 107).

## Supporting information



Supporting Information

## Data Availability

Samples of the data/code that support the findings of this study are included with this submission.

## Appendix 1

### Model structures

**Model 1.1/2.1 Average viewing time (log corrected) per panel**

*log10Viewing Time∼ Condition*Contains Text + Expertise + (1 | Subject) + (1 |Comic)*

**Model 1.2 Landing position of first fixation in next panel (Text or image)**

*LandingPosition∼ Condition + Expertise + (1 | Subject) + (1 |Comic)*

**Model 1.3 Proportion of first pass skips of panels**

*FirstPassSkip∼ Condition + Contains Text + Expertise + (1 | Subject) + (1 |Comic)*

**Model 1.4/2.4 Proportion of regressions launched**

*Regression∼ Condition* Contains Text + Expertise + (1 | Subject) + (1 |Comic)*

**Model 2.2 Landing position of first fixation in next panel (Text or image)**

*LandingPosition∼ Condition*Text Location + Expertise + (1 | Subject) + (1 |Comic)*

**Model 2.3 Proportion of first pass skips of panels**

*FirstPassSkip∼ Condition*Contains Text + Expertise + Neighboring Panel + (1 | Subject) + (1 |Comic)*

## Appendix 2

### Experiment 1 model summaries

Model 1.1: Model to predict average viewing time per panel

**Table:**

Fixed effects	Estimate	*SE*	*t*	***p***
(Intercept)	3.22	0.053	60.56	<0.001
Control versus *n*	0.013	0.015	11.39	<0.001
Control versus *n* + 1	0.018	0.011	1.55	0.122
Control versus *n* + 2	0.012	0.015	1.07	0.283
Contains Text	0.135	0.013	10.52	<0.001
Text*Condition(*n*)	−0.046	0.011	−4.02	<0.001
Text*Condition(*n* + 1)	−0.003	0.011	−0.24	0.810
Text*Condition(*n* + 2)	0.001	0.011	0.05	0.958
Expertise	−0.002	0.011	−0.45	0.657
Random effects Variance				
Subjects	0.023			
Items	0.005			

Model 1.2: Model to predict proportion of first fixations to text or image regions in next panel

**Table:**

Fixed effects	Estimate	*SE*	*z*	***p***
(Intercept)	0.962	0.304	3.16	0.002
Control versus *n*	−0.532	0.136	−3.92	<0.001
Control versus *n* + 1	−0.321	0.138	−2.33	0.020
Control versus *n* + 2	−0.185	0.138	−1.34	0.180
Expertise	−0.013	0.022	−0.58	0.563
Random effects Variance				
Subjects	0.506			
Items	0.236			

Model 1.3: Model to predict proportion of first pass skips of panels

**Table:**

Fixed effects	Estimate	*SE*	*t*	***p***
(Intercept)	−4.61	0.587	−7.86	<0.001
Control versus *n*	0.606	0.306	1.98	0.048
Control versus *n* + 1	0.409	0.316	1.30	0.195
Control versus *n* + 2	0.006	0.340	0.016	0.987
Contains Text	0.169	0.109	1.56	0.120
Expertise	0.012	0.049	0.240	0.810
Random effects Variance				
Subjects	1.86			
Items	<0.001			

Model 1.4: Model to predict proportion of regressions to previous panels

**Table:**

Fixed effects	Estimate	*SE*	*z*	***p***
(Intercept)	−1.19	0.242	−4.91	<0.001
Control versus *n*	−1.07	0.105	−10.15	<0.001
Control versus *n* + 1	−0.785	0.090	−8.72	<0.001
Control versus *n* + 2	−0.806	0.093	−8.68	<0.001
Contains Text	0.131	0.041	3.17	0.002
Text*Condition(*n*)	0.159	0.105	1.52	0.129
Text*Condition(*n* + 1)	0.130	0.090	1.45	0.146
Text*Condition(*n* + 2)	0.060	0.092	0.654	0.513
Expertise	0.016	0.019	0.864	0.387
Random effects Variance				
Subjects	0.310			
Items	0.011			

## Appendix 3

### Experiment 2 model summaries

Model 2.1: Model to predict average viewing time per panel

**Table:**

Fixed effects	Estimate	*SE*	*t*	***p***
(Intercept)	3.18	0.091	35.05	<0.001
Control versus *n*	0.130	0.014	9.43	<0.001
Control versus *n* + 1	0.031	0.014	2.28	0.023
Contains Text	0.210	0.006	36.74	<0.001
Text*Condition(*n*)	−0.076	0.014	−5.55	<0.001
Text*Condition(*n* + 1)	−0.039	0.014	−2.83	0.005
Expertise	0.007	0.003	2.23	0.034
Random effects Variance				
Subjects	0.014			
Items	0.040			

Model 2.2: Model to predict proportion of first fixations to text or image regions in next panel

**Table:**

Fixed effects	Estimate	*SE*	*z*	***p***
(Intercept)	0.296	0.301	0.983	0.326
Control versus *n*	−0.804	0.092	−8.768	<0.001
Control versus *n* + 1	−0.100	0.087	−1.15	0.252
Text location	−0.756	0.074	−10.13	<0.001
Expertise	−0.011	0.012	−0.958	0.338
Random effects Variance				
Subjects	0.154			
Items	0.629			

Model 2.3: Model to predict proportion of first pass skips of panels

**Table:**

Fixed effects	Estimate	*SE*	*z*	*p*
(Intercept)	−1.67	0.343	−4.88	<0.001
Control versus *n*	−0.719	0.146	−4.92	<0.001
Control versus *n* + 1	−1.20	0.169	−7.11	<0.001
Contains Text	0.361	0.10	3.72	<0.001
Text*Condition(*n*)	−0.106	0.145	−0.73	0.464
Text*Condition(*n* + 1)	−0.500	0.168	−2.98	0.003
Neighboring Panel	0.170	0.111	1.53	0.125
Expertise	−0.013	0.022	−0.60	0.547
Random effects Variance				
Subjects	0.554			
Items	0.202			

Model 2.4: Model to predict proportion of regressions to previous panels

**Table:**

Fixed effects	Estimate	*SE*	*z*	***p***
(Intercept)	−1.43	0.154	−9.28	<0.001
Control versus *n*	−0.691	0.104	−6.67	<0.001
Control versus *n* + 1	−0.664	0.094	−7.05	<0.001
Contains Text	−0.099	0.046	−2.15	0.031
Text*Condition(*n*)	−0.212	0.104	−2.04	0.041
Text*Condition(*n* + 1)	−0.115	0.095	−1.22	0.223
Expertise	0.001	0.009	0.16	0.873
Random effects Variance				
Subjects	0.068			
Items	0.063			
