# Supplementary material for: Coming Up Next: The Extent of the Perceptual Window in Comic Reading
Source: Cogn Sci. 2025 Nov 19;49(11):e70142. doi: 10.1111/cogs.70142 (PMC12631053; doi:10.1111/cogs.70142)
Supplement: Supplementary file 1 — Supporting Information [file COGS-49-e70142-s001.docx]

Supplementary Material

Experiment 1: Reduction in first-pass skipping over trials

**Table 2a.***Trial number influencing average proportion of first-pass skipping behaviours across conditions*

| *Fixed Effects* | *Estimate* | *SE* | *t* | *p* |
| --- | --- | --- | --- | --- |
| (Intercept) | -3.44 | 0.607 | -5.66 | <0.001 |
| Trial | -0.094 | 0.015 | -6.42 | <0.001 |
| Control vs. *n* | 1.43 | 0.536 | 2.67 | 0.008 |
| Control vs. *n+1* | 0.983 | 0.558 | 1.76 | 0.078 |
| Control vs. *n+2* | -0.064 | 0.624 | -0.10 | 0.919 |
| Trial*Condition (*n)* | -0.089 | 0.042 | -2.10 | 0.036 |
| Trial*Condition (*n+1)* | -0.048 | 0.039 | -1.23 | 0.218 |
| Trial*Condition (*n+2)* | 0.005 | 0.040 | 0.134 | 0.893 |
| Random effects Variance |  |  |  |  |
| Subjects | 1.87 | | | |
| Items | <0.001 | | | |

A significant interaction was found between the trial number and the condition for condition *n* only- as trial number increased, the amount of first-pass skipping reduced. Thus, this is a plausible explanation for increased skips in the condition with the least peripheral information available to readers.
